# Editorial: The Neuroethology of Social Behavior

**DOI:** 10.3389/fncir.2022.897273

**Published:** 2022-05-09

**Authors:** Gervasio Batista, Joel D. Levine, Ana Silva

**Affiliations:** ^1^Department of Molecular and Cellular Biology, Harvard University, Cambridge, MA, United States; ^2^Department of Ecology and Evolutionary Biology, University of Toronto Mississauga, Toronto, ON, Canada; ^3^Laboratorio de Neurociencias, Facultad de Ciencias, Universidad de la República, Montevideo, Uruguay

**Keywords:** neuroethology, social behavior, neuroscience, evolution, neural mechanism

One of the great challenges in modern biology is to understand how social behaviors arise through a combination of genetic and environmental factors, and how they are implemented by brain circuits. Increasingly, studies in the field and laboratory have shown that group dynamics are critical to the life of individuals and that wherever groups are observed, behavior is modulated by individual members as well as by the presence of others. We pursued this Frontiers Research Topic to seek Neuroethological perspectives on principles of neural circuits and social behavior across organisms and fields.

Neuroethology takes advantage of species diversity to study the neural underpinnings of natural behaviors. Each organism provides unique answers to diverse long-standing problems that are fundamental to understanding brain function and social processes. Invertebrates, for instance, have been pivotal in tracking the genetic basis of social cohesion. Newborn birds have increasingly become ideal systems to untangle *nature vs. nurture* questions, given their innate responses to social stimuli and precocious learning capacities. The well-understood circuits controlling vocal and electric communication in fish shed light on the architecture of social signals. Field studies in frogs have forged new evidence on the molecular basis of social behavior and the origins of its natural variability. The present collection of articles represents the diversity of theoretical and experimental approaches necessary to establish a compelling view of social behaviors. From flies to humans, authors stage the richness of studying social phenomena at multiple levels, from groups of individuals to genes, and confirm the importance of picking the right organisms to address specific questions.

Early life experience determines brain maturational trajectories that affect a number of social and non-social behaviors. Similarly, parental care has long-lasting effects on progeny as Zeng et al. exemplify. Autry and O'Connell discuss and compare behavior and brain approaches to tackle generalizable and distinctive principles in parenting. A major point raised by the authors is that parental care strategies across organisms need to be understood in terms of their ecological challenges. In turn, field studies become an important aspect of information in understanding parenting across species. In laboratory settings, the continuously growing toolbox for neuronal manipulation allows the resolution of specific circuits driving parental care. As argued by Autry and O'Connell it is at the crossroads of ethological and neurogenetic inroads that future avenues will open.

Whether brains are innately tuned to social stimuli has been elusive to experimental inquiry. Newborn chicks, extensively used in filial imprinting studies, have been previously shown to be attracted to face-like visual stimuli and patterns of biological motion. Lorenzi et al. report that the chicks' preference toward animated objects is controlled by thyroid hormones. In addition, Adiletta et al. demonstrated that embryonic exposure to Valproic Acid (an agent used to model autistic-like behaviors in preclinical studies) disrupts the innate orienting behaviors toward face-like stimuli. Thus, Adiletta et al. argue that chicks might be useful to model face processing deficits in ASD.

Two teleost model systems, weakly electric fish and vocal fish, stand out for their contributions to the understanding of the neuroendocrine basis of social communication. These distantly related teleost groups that produce either vocalizations or electric organ discharges have been traditionally compared. Dunlap et al. discuss an updated view on the behavioral neuroendocrinology of vocal and electric fish and offer complementary insights into social communication biology. Dunlap et al. revise recent findings in both teleost systems and make direct comparisons to highlight how these analogous communication systems have evolved similar and different mechanisms.

Weakly electric fish have also recently emerged as advantageous model systems for the study of complex social behaviors, in which electric signaling is part of their displays. The extensive knowledge of electrocommunication set the stage for more complex evolutionary comparisons based on social behavioral strategies. Both song sparrows and banded knifefish display territorial aggression uncoupled from reproduction. Quintana et al. summarize recent findings on the neuromodulation of non-breeding aggression in fish and birds to establish general principles in the regulation of social behavior. Notably, neurosteroids (steroids synthesized locally in the brain) and neuropeptide Y play important roles in non-breeding aggression across organisms.

The advanced genetics of *Drosophila melanogaster* and broadly available tools to dissect specific circuits governing behaviors puts this organism at a privileged site to investigate the basis of sociability. However, flies have been considered solitary for decades. The recent use of social network analysis has revealed that groups of flies present a structure rooted in their genes. Jezovit et al. review several studies to highlight methodological differences and common findings to inspire new ideas in the field of fly social networks.

While human social interactions are difficult to translate into animal models, complex social traits have been successfully replicated in preclinical studies. Leong et al. describe how social learning and its underlying inter-brain synchrony can be modeled in mice. In such settings, the authors argue that optogenetic manipulation of social dyads might open novel avenues for future studies. On the other hand, the authors discuss major caveats to this approach to promote discussion in the field. Li et al. offer their view on how animal models can be useful in discovering novel therapeutic strategies for social deficit disorders such as autism and schizophrenia.

In an era where cutting-edge experimental tools are available across taxa, the scientific community needs to embrace comparative biology as a keystone of future studies of social behaviors. As we understand the peculiarities of species-specific social behaviors, an evolutionary perspective will grant us new experimental paradigms and theoretical concepts to extract common principles linking brain function and social performance. From an evolutionary perspective sexual reproduction, communications, group structure, and problem-solving are important problems that need to be understood and explained. We believe that the papers in this Frontiers Research Topic will drive more conversations about this important issue and invite researchers from around the world to adopt the neuroethological approach to the study of social behavior.

## Author Contributions

GB, JL, and AS wrote and edited the article. All authors contributed to the article and approved the submitted version.

## Conflict of Interest

The authors declare that the research was conducted in the absence of any commercial or financial relationships that could be construed as a potential conflict of interest.

## Publisher's Note

All claims expressed in this article are solely those of the authors and do not necessarily represent those of their affiliated organizations, or those of the publisher, the editors and the reviewers. Any product that may be evaluated in this article, or claim that may be made by its manufacturer, is not guaranteed or endorsed by the publisher.

